# Actinomycosis

**Published:** 1896-04

**Authors:** J. L. Wingate

**Affiliations:** Student


### ACTINOMYCOSIS.[1]

BY J. L. WINGATE,
STUDENT.

THE subject of my essay to-night is one which has excited a great deal of interest all over the world, and at the present time there is considerable difference of opinion among some of our great scientists, but one fact is quite clear to us all, that actinomycosis, like so many other of our contagious forms of disease, is proved to be caused by the existence of micro-organisms.

During the last ten years great discoveries have been made in bacteriology, or the germ theory, as it is sometimes called. For this knowledge we owe much to such able men as Pasteur, Graves, Nocard, Darwin, and many others. I read with great interest a little bacteriological sketch on *Zymosis and Pathogenesis*, in which, among other topics connected with bacteriology, was the history of M. Pasteur's early life and the way in which he achieved some of his great discoveries with the microscope. For instance: In France, in 1853, the silk-culture was becoming very much impoverished by a contagious disease among the

[1] Read before the Veterinary Medical Association, Ontario Veterinary College, October 25, 1895.

worms, known as "pebrine." Every conceivable means had been tried to check it without success, the government then called on M. Pasteur to make investigations, and he, after many months of diligent work, succeeded in finding out the cause and was able to restore to France one of her greatest industries.

Professor Charcot, the neurologist, says: "Art without science is apt to degenerate into routine;" and another remark of his is: "The hackneyed skepticism which people so willingly oppose to all progress of the human mind is a comfortable pillow for lazy heads, but the period in which we live allows no time for falling asleep, when every hour must sweat her sixty minutes to the death."

To return more particularly to the disease in question, actinomycosis is communicable to man, therefore the public health is endangered by its existence. For this reason alone the disease becomes one of importance, especially to us as members of the profession, because some time during our practice we are sure to be called to one of these cases and our opinion asked as to whether the flesh of such an animal is fit for human food. Who should be more competent than the veterinary surgeon in this work of examining meat killed for human food? But some people say it should be the medical man that should make these examinations; why, I cannot conceive. What does a medical man know about the lower animals? What should he know? No, gentlemen, it is decidedly in our department, but it remains with each and every one of us to keep the profession at the proper standard by advancing with the progressive age in which we live; if we do this our results will overthrow such prejudices, proving we are not behind the age in this particular branch of our work as is so often supposed.

As recently as the middle of the present century actinomycosis was thought to be a form of "scrofulæ," or another name for it was "clyers." In 1863 Rivolta discovered in the diseased tissues the existence of the rayed fungus, but it was not until 1877 that Bollinger discovered the constant presence of this low form of bacteria, which is now known to be the vegetable parasite actinomyces.

This disease is peculiar to cattle, although it is occasionally seen in the hog and man. It is caused, as I have just mentioned, by the vegetable parasite actinomyces. Of the life-history of this parasite very little has been discovered up to the present time, but it is generally believed that the animal becomes infected through its food; whether the source is any particular plant or group of plants, or whether it is from the nature of the soil, still remains a mystery. It is claimed by a veterinarian in Denmark that grain or hay grown and harvested on land recently reclaimed from the sea will cause the disease. Barley, barley-straw, rye, Indian corn, seem to be the most common medium by which the disease is communicated to cattle, but an animal may become infected, or, more properly speaking, inoculated, through any of the natural orifices of the body.

Take first the most common and worst form of this disease, that effecting the head, sometimes called "lump-jaw," "big-head," "wooden-tongue." Here we have the fungus carried into the mouth by means of the food; the mucous membrane may become pricked during mastication by the sharp points of the straw, or, as is commonly the case, by the sharp spikelets found on the clevels of barley; thus an entrance is made for the fungus and the seeds sown of this loathsome disease. Again it is said by some that dentition plays an active part in giving access to the fungus. The eruption of the teeth in the ox commences at two years, and the full mouth is attained at four years or a little more, so we may say that any time between the age of two and five years the teeth are in a constant state of eruption; during this time the fungus has a splendid opportunity for gaining access to the parts. When we consider that the bones of the jaws are more often affected than any other part of the animal, this explanation seems very satisfactory. Another point which helps to strengthen this theory is, that young animals are more often affected than the old ones.

The enemy installed, we will now follow the development of the disease. In the early stages its progress is very slow; it may be a year or two before the animal shows any of the characteristic symptoms; but the disease slowly but surely develops according to the existing conditions. An affected animal will first cease feeding, and consequently fall away in condition; if a milch cow, she will go off her milk and show the general symptoms of disease. The jaws will begin to enlarge according to the situation of the tumors, and an examination of the mouth shows the teeth to be involved, becoming loose and interfering materially with mastication. If allowed to go on the tumors will attain, in some cases, an enormous size, sometimes as large as a man's head; finally reaching the suppurating stage, and terminating in an open sore from which there will be a continuous discharge of pus.

I have been speaking more particularly of that form of the disease affecting the jaw-bones, but it must not be forgotten that any part of an animal may be attacked; sometimes the seat of the disease is the soft structures of the head, involving the tongue, larynx, pharynx, salivary glands, more especially the parotid gland. The tongue in this case becomes very much swollen and as hard as wood, hence the term "wooden-tongue" which has been applied to it (this form of the disease being the one most prevalent in England); or the lungs may be extensively diseased, but rarely the intestines. It is not uncommon to find the umbilucus involved. The tumors have a special tendency to work toward the mucous and cutaneous surfaces. The disease is sometimes mistaken for tuberculosis, but an examination of the tumor, wherever it is located, with a common pocket-lens will always show the ray-fungus to be present in actinomycosis.

For treatment the internal administration of iodide of potassium has proved quite a specific. This is well shown by the experiments made at Chicago a short time ago, where 185 animals suffering from this disease in all its stages were quarantined under this treatment; in some the tumors had attained a great size. The whole were slaughtered and carefully examined; out of the total number, 71 per cent. were found cured; those showing internal lesions were 7, or 3.8 per cent. of the animals under experiment. It must be remembered that a considerable number of these animals had the disease in the advanced stages, so the results on a whole were very good. If the disease is taken in hand during the early stages I think it will be safe to say that 90 per cent. can be cured. It still remains a mystery in what this specific effect consists which iodide of potassium has on the actinomyces fungus, but we know it has great absorbing powers when given internally, especially on all pathological accumulation of cells. In connection with protoplasm, carbonic acid, and water, the iodine will become free and combine with the cells of the pathological product, which will decrease their vital power and facilitate their absorption. From the fact that the iodine salts are rapidly absorbed by all glandular structures, it would not be advisable to give iodide of potassium to milch cows as it would reduce the milk-secretion or stop it altogether; furthermore, a great part of the medicine is excreted through the milk, making it unfit for food. This drug permeates all the tissues of the body, including the muscles and bones, but is rapidly excreted again through the urine and milk. Care must be taken in the use of this medicine, because, even when given in proper doses, after a certain time the iodine will not only affect the diseased tissues, but combine with the normal cells in the body and produce serious symptoms called iodism; these are general emaciation, atrophy of lymphatic and mammary glands, combined with chronic catarrh of the nose and conjunctiva, and desquamation of the epidermis; if large doses have been given catarrh of stomach and intestines may be produced, and even hemorrhages. The feces will be hard and dry, coated with mucus or perhaps blood.

The dose of iodide of potassium for an averaged-sized animal is ʒij–ʒiij twice a day until the first symptoms of iodism are manifested, which will be from one week to ten days; it should be at once stopped for two or three days and a dose of magnesia sulphate given, then the patient is ready for another week's treatment, and so on until recovery.

Post-mortem examination shows the actinomyces in small masses, which are easily seen by the naked eye; they are white to yellowish in color and of hard consistency, due to formation of fibrous tissue. Sometimes they are gritty in character. Take one of the largest masses, and it looks very much like a handful of rice would deposited in a heap. The masses are club-like in shape, radiating from the centre to the circumference of the tumor, the external surface consisting of the rounded thick ends. Suppuration commences in the deep parts of the tumor, then the elements become indistinct, forming an amorphous mass, which in time may become calcified; the ray appearance always remains on the surface. The fungus first develops in giant-cells surrounded by fibrous tissue; thus the definiteness of the growth of the tumor is seen from the surface to the centre. When it is a bony structure that is involved the swollen bone becomes arranged in bands, the spaces between being filled up with a creamy pus-like substance of a gelatinous character, giving it a spongy appearance. These growths commence first in the medullary portion, gradually working to the external surface. In this tissue the parasite seems to thrive luxuriantly, consequently we find the bones of the jaw and the tongue most often affected. Sometimes we have cold abscesses instead of hard tumors, but they never attain a great size, and the parasite is found intermingled with the pus which the abscess always contains. When the disease attacks man there is a great tendency to the formation of these abscesses, with considerable necrosis of tissue, often proving fatal through pyæmia resulting.

I must give a few lines on prevention before I close. Knowing that animals contract this disease principally through the medium of their food, much has to be discovered of the life-history of the parasite before any satisfactory measures can be expected on this score; but not infrequently man has contracted the disease by the habit some people have of picking up a piece of straw and chewing it, so we can take a warning here. From what I have said in the previous part of my essay concerning the way in which the fungus enters the system, I think you agree with me that the disease is inoculable rather than contagious; hence animals suffering from actinomycosis should be isolated and attendants should be warned to be careful, especially if the case has gone on to suppuration, not to get the discharge into any open wounds they may have on their hands; and last, but not least, is the careful inspection of our stockyards and slaughter-houses. This parasite, like all others, is destroyed by sufficient cooking; but even this does not warrant eating the meat of affected animals. Therefore it is a question whether all animals suffering from actinomycosis should not be rejected as unfit for food. Cattle are plentiful and comparatively low in price, and the preservation of health and prevention of disease in man must be considered at any cost. This last statement may seem rather a bold one to make, but I have an article from one of the medical journals which justifies what I have said.

